# Reuse of single-dose nevirapine in subsequent pregnancies for the prevention of mother-to-child HIV transmission in Lusaka, Zambia: A cohort study

**DOI:** 10.1186/1471-2334-8-172

**Published:** 2008-12-30

**Authors:** Jan Walter, Louise Kuhn, Chipepo Kankasa, Katherine Semrau, Moses Sinkala, Donald M Thea, Grace M Aldrovandi

**Affiliations:** 1 Childrens Hospital Los Angeles, University of Southern California, Los Angeles, CA, USA; 2 Gertrude H. Sergievsky Center and Department of Epidemiology, Mailman School of Public Health, Columbia University, New York, NY, USA; 3 University Teaching Hospital, University of Zambia, Lusaka, Zambia; 4 Center for International Health and Development at the Boston University School of Public Health, Boston, MA, USA; 5 Lusaka District Health Management Team, Lusaka, Zambia

## Abstract

**Background:**

Single-dose nevirapine (SDNVP) for the prevention of mother-to-child HIV transmission (PMTCT) results in the selection of resistance mutants among HIV-infected mothers. The effects of these mutations on the efficacy of SDNVP use in a subsequent pregnancy are not well understood.

**Methods:**

We compared risks of perinatal HIV transmission between multiparous women who had previously received a dose of SDNVP (exposed) and those that had not (unexposed) and who were given SDNVP for the index pregnancy within a PMTCT clinical study. We also compared transmission risks among exposed and unexposed women who had two consecutive pregnancies within the trial. Logistic regression modeling was used to adjust for possible confounders.

**Results:**

Transmission risks did not differ between 59 SDNVP-exposed and 782 unexposed women in unadjusted analysis or after adjustment for viral load and disease stage (adjusted odds ratio 0.6, 95% confidence interval [CI] 0.2 to 2.0). Among 43 women who had two consecutive pregnancies during the study, transmission risks were 7% (95% CI 1% to 19%) at both the first (unexposed) and second (exposed) delivery. The results were unchanged, if infant death was included as an outcome.

**Conclusion:**

These data suggest that the efficacy of SDNVP may not be diminished when reused in subsequent pregnancies.

## Background

Single-dose nevirapine (SDNVP) given at the onset of labor to the mother and within 72 hours of birth to the infant is a safe, efficacious, simple and cost-effective intervention for the prevention of mother-to-child HIV transmission (PMTCT) [1]. It has been widely implemented in resource-constrained countries, where more complex and potent regimens are not available [2].

A drawback of this regimen is the selection of resistance mutations in a large proportion of exposed women and their infected infants [2]. These mutations may reduce virologic susceptibility to treatment with non-nucleoside reverse transcriptase inhibitor-based regimens if initiated soon after delivery [3,4]. The mutations may also remain detectable in a small proportion of women at above pre-exposure levels as late as 1 year post-partum [5-7], raising concerns that use of this regimen for PMTCT in subsequent pregnancies compromises the efficacy of the intervention.

Studies from South Africa/Cote d'Ivoire [8] and Uganda [9] have found no decrease in SDNVP efficacy during subsequent pregnancies. However, these studies included few subtype C infected women, who may have a higher frequency of resistant strains after exposure to SDNVP [10]. The predominant subtype in circulation in Zambia is C. We compared the transmission risk associated with use of SDNVP in repeat pregnancies among women enrolled in a clinical PMTCT trial in Zambia [11]. Among study participants with at least one previous live birth (multiparous) who were given SDNVP for the study, we compared the perinatal HIV transmission risk between women who reported previous SDNVP use and those who did not. In a second analysis, we compared transmission risks between the first (unexposed) and second (SDNVP-exposed) delivery of a subset of women who had two consecutive pregnancies within the study.

## Methods

### Study procedures

Women were enrolled in the Zambia Exclusive Breastfeeding Study at two antenatal clinics in Lusaka, Zambia, between April 2001 and May 2004 [11,12]. All women were given a 200 mg tablet of nevirapine to be taken at the onset of labor. At delivery, women were asked if and when they had consumed the nevirapine tablet. If more than 48 hours had passed or they had not taken it, they were given a second dose. The newborn received 0.6 ml nevirapine suspension as soon as possible after the delivery. All women were counseled to exclusively breastfeed until at least four months. As part of prenatal care, all women were offered multivitamins, presumptive malaria prophylaxis and starting in November 2003 cotrimoxazole prophylaxis if enrolment CD4 cell counts were below 200 cells per μl [13]. Antiretroviral treatment only became available in late 2004, towards the end of the study.

Maternal blood samples were collected at enrolment, CD4 counts (BD Immunocytometry Systems, San Jose, CA) and plasma viral load (Roche Amplicor^® ^version 1.5, Branchburg, NJ) were determined. Women were asked about HIV-related clinical conditions and WHO clinical stage was assigned based on these self-reports. To account for pregnancy-related weight gain, any weight loss was categorized as severe. Weight and height was measured and Body Mass Index (BMI) calculated as kg per square meter. Infant dried blood spots were collected at delivery, one week, one month, at least monthly until 6 months of age and then every 3 months until the end of the follow-up at 24 months and tested for HIV DNA by polymerase chain reaction (PCR) as described previously [11].

The study was approved by the Institutional Review Boards of the investigators' institutions and all participants provided written informed consent.

### Statistics

HIV transmission events were included in the analysis if they occurred within 42 days post-partum to allow for variation in the time of the 1 month HIV test. Timing of HIV transmission was categorized by the PCR test results as intrauterine (infant PCR positive within 3 days of delivery), or intrapartum/early breastfeeding (between 4 and 42 days postpartum). For multiple births, the first live-born infant was included in the analysis. Birth weight of infants born at home was only included in the analysis if the infants were presented at the clinic within 3 days after delivery.

We used the chi-square test, or if the expected cell size was below 5 we used Fisher's Exact Test, to compare categorical variables, Cochran-Armitage Trend Test for ordered categorical variables, the T-test to compare normally-distributed continuous variables and the Wilcoxon Rank Sum Test to compare non-normally-distributed continuous variables. Logistic regression modeling was conducted to adjust for possible confounding. Consecutive deliveries of women during the study were compared using paired T-tests for normally distributed variables, the Wilcoxon Signed Rank Test for non-normally distributed continuous paired variables, and the McNemar Test for categorical variables. Asymptotic p-values were calculated for McNemar Test, unless the expected cell counts were less than 5 in which case, exact values were calculated. Exact binomial confidence intervals were calculated for the transmission risks among women with two deliveries.

All statistical analyses were performed using SAS software (Version 9.1, Cary, NC).

## Results

### Study population

**Figure 1:**
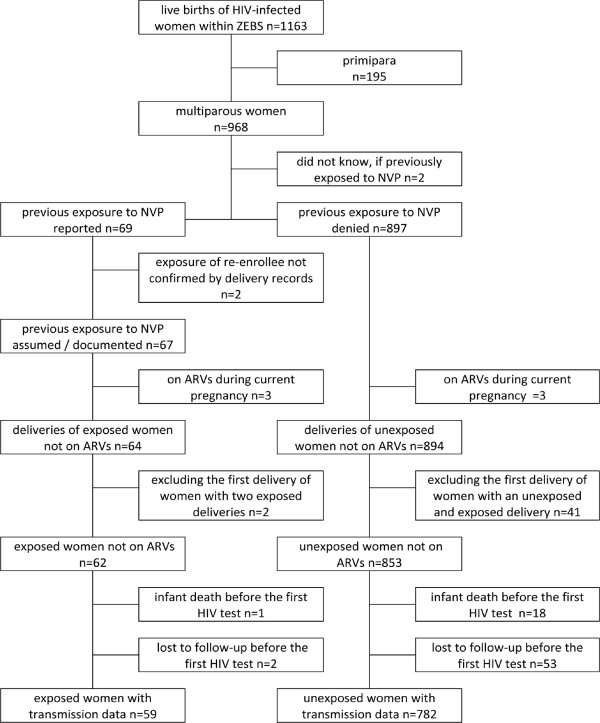
**Repeated SDNVP exposure within the Zambia Exclusive Breastfeeding Study and selection of the study cohort**.

Figure 1 illustrates the selection of the study cohort. We excluded primiparous women, women who did not know if they had been exposed to SDNVP, women with a self-reported exposure that contradicted the study documentation, as well as women on antiretroviral treatment during pregnancy. To assure statistical independence of the groups, we further limited the study population to one delivery (i.e. the second) per woman, resulting in the inclusion of 62 deliveries of SDNVP-exposed and 853 of unexposed women. Similar frequencies of infants died (1 (2%) versus 18 (2%), p = 1.0) or were lost to follow-up (2 (3%) versus 53 (6%), p = 0.58) before their HIV-status could be determined, resulting in a final study cohort of 59 exposed and 782 unexposed women.

Of the 59 exposed women, 45 (76%) had received nevirapine at a preceding delivery within our study, nine (15%) reported participation in an earlier study at the same site and for 5 (8%) the source of exposure was unknown.

Unexposed women had a higher viral load and more advanced disease stage (≥ stage III) at enrolment than SDNVP-exposed women. They also had fewer previous live births, lower BMI, and tended to have infants of lower birth weight. CD4 counts were not significantly different (Table 1).

**Table 1:** Characteristics HIV-infected multiparous mothers and their infants by previous exposure to single-dose nevirapine (SDNVP).

	Mother previously nevirapine exposed	Mother previously nevirapine un-exposed	p-value
N^a^	59	782	
Socio-demographic variables			
Maternal age [mean ± SD]	27.3 ± 5.1	26.9 ± 4.9	0.59
Number of previous live births [median (IQR)]	3 (2, 4)	2 (1, 3)	0.001
HIV-related factors at enrolment			
HIV viral load [copies per ml ]			
Median (IQR)	20,501 (7,001; 60,617)	39,401 (9,728; 130,058)	0.04
< 10 000 [N (%)]	17 (29)	202 (26)	
10 000–99 999 [N (%)]	31 (53)	346 (44)	
≥ 100 000 [N (%)]	10 (17)	232 (30)	0.11 (trend)
CD4 cells [counts per μl]			
Median (IQR)	335 (222, 453)	316 (190, 468)	0.86
< 200	14 (24)	205 (26)	
200–349	19 (33)	238 (30)	
≥ 350	25 (43)	338 (43)	0.86 (trend)
N (%) WHO clinical stage III	11 (19)	337 (43)	0.0002
BMI at 1 month post partum [mean ± SD]	22.7 ± 3.2	21.7 ± 3.4	0.03
Hemoglobin [g/dl] [mean ± SD]	10.9 ± 1.5	10.6 ± 1.5	0.12
Positive rapid plasma regain test for syphilis [N (%)]	6 (11)	135 (18)	0.15
Obstetric and infant variables			
Caesarean sections [N (%)]	1 (2)	14 (2)	1.0
Low birth weight [N (%) < 2500 g]	2 (4)	83 (11)	0.07
Birth weight [mean ± SD]	3077 ± 439	3015 ± 496	0.36
Preterm delivery [N (%) ≤ 34 weeks of gestation]	11 (19)	178 (23)	0.50
Sex [N (%) male]	26 (44)	407 (52)	0.23
Nevirapine prophylaxis at current delivery			
Maternal dose taken [N (%)]	58 (98)	745 (95)	0.51
Infant dose taken [N (%)]	57 (98)	733 (95)	0.35
HIV transmission and infant mortality^b^			
Infants HIV positive ≤ day 42 [N (%)]	3 (5)	94 (12)	0.11
Infants HIV positive or died ≤ day 42 [N (%)]^c^	4 (7)	127 (16)	0.06

a) numbers may differ from the total due to missing data

b) Loss to follow-up at 42 days (as defined as HIV-uninfected, did not die ≤ 42 days, but was last tested before the one month visit) was not significantly different between both groups (1 (2%) versus 63 (8%), p = 0.08).

c) The study size for the combined analysis of infant mortality and HIV transmission was slightly larger as it includes 1 infant in the exposed and 18 in the unexposed group who died before the HIV status was established.

### HIV transmission risks between SDNVP-exposed and unexposed

The risk for perinatal HIV transmission and the risk for combined HIV transmission and infant death ≤ 42 days post-partum did not significantly differ by maternal exposure to nevirapine and was slightly lower in the exposed group (exposed: 3 (5%) versus unexposed: 94 (12%), p = 0.11) (Table 1). The risks were also similar if further stratified by the probable timing of HIV transmission: intrauterine – 1 (2%) versus 49 (6%), p = 0.25 – and intrapartum/early breastfeeding – 2 (3%) versus 45 (6%), p = 0.57 in exposed versus unexposed deliveries respectively.

There continued to be no association between maternal SDNVP exposure status and transmission after adjusting for confounding by logistic regression. In these models, the adjusted odds ratio (AOR) for HIV transmission ≤ 42 days post-partum among exposed relative to unexposed was 0.6 (95% CI: 0.2 to 2.0) after adjusting for maternal viral load (AOR 3.0, 95% CI 2.1 to 4.2 per log10 increase) and maternal clinical stage (AOR: 1.8, 95% CI 1.1 to 2.8). None of the other factors listed in Table 1 was independently associated with the transmission risk if added to the above model, or substantially changed the estimate of the nevirapine exposure effect. The results were similar, if the combined risk for HIV transmission and infant death was chosen as the outcome.

### Women with two deliveries within the study

Of the 45 women who had two live births during the study, 2 reported a previous exposure to SDNVP at their first enrolment. One of them transmitted HIV to their infant at the second exposed delivery (the first within the study), however neither of them transmitted at the third exposed delivery. We next compared transmission risks between the remaining 43 women with an unexposed and a subsequently exposed delivery.

The median time between the two deliveries was 22 months (range 12 to 42 months, IQR: 17 to 31 months), during which the median CD4 cell count declined by 96 cells per μl. There was no significant difference in the viral load between pregnancies (Table 2) with only 4 women decreasing by more than 0.7 log_10 _copies per ml and 4 increasing by more than 0.7 log_10 _copies per ml. In most of the other characteristics the women were comparable at their first and second pregnancy (Table 2).

**Table 2:** HIV transmission among 43 HIV-infected women who had two deliveries within the study.

	Mother previously nevirapine un-exposed (first delivery)	Mother previously nevirapine exposed (second delivery)	p-value
Socio-demographic variables			
Maternal age [mean ± SD]	25.3 ± 4.7	27.3 ± 4.9	< 0.0001
Primipara [N (%)]	5 (12)	0 (0)	/
HIV-related factors at enrolment			
HIV viral load [median copies per ml (IQR)]	30,412 (6,864; 80,557)	18,918 (7,162; 78,152)	0.34
CD4 cells per μl [median (IQR)]	430 (303, 595)	334 (188, 460)	< 0.0001
Disease stage III [N (%)]	14 (33)	9 (21)	0.30
BMI at 1 month post partum [mean ± SD]	21.4 ± 2.8	22.4 ± 3.1	0.007
Hemoglobin [g/dl] [mean ± SD]	10.7 ± 1.5	11.0 ± 1.4	0.24
Positive rapid plasma regain test for syphilis [N (%)]	9 (22)	5 (12)	0.22
Obstetric and infant variables			
Caesarean sections [N (%)]	2 (5)	0 (0)	/
Low birth weight [N (%) < 2500 g]	5 (12)	2 (5)	0.45
Birth weight [mean ± SD]	3029 ± 536	3042 ± 469	0.82
Preterm delivery [N (%) ≤ 34 weeks of gestation]	5 (12)	8 (19)	0.75
Sex [N (%) male]	23 (53)	16 (37)	0.14
Nevirapine prophylaxis at current delivery			
Maternal dose taken [N (%)]	43 (100)	43 (100)	/
Infant dose taken [N (%)]	40 (93)	41 (98)	1.0
HIV transmission and infant mortality			
Infants HIV positive ≤ day 42 [N (%)]	3 (7)	3 (7)	1.0
Infants HIV positive or died ≤ day 42 [N (%)]	4 (9)	3 (7)	1.0

Numbers may differ from the total due to missing data

The risk for HIV transmission did not differ between the SDNVP-naive and exposed pregnancies (3 (7%), 95% CI: 1% to 19% in both groups, p = 1.0) with similar numbers of intrauterine (0 versus 1) and intrapartum/early breastfeeding transmissions (3 versus 2) (Table 2). These six transmissions occurred to 5 different women, as one mother transmitted HIV in both deliveries (intrapartum/early breastfeeding at first and intrauterine at the second).

## Discussion

In this well-defined cohort of HIV-infected women in Lusaka, Zambia, the reuse of SDNVP for PMTCT at a subsequent pregnancy did not reduce the efficacy of the intervention. Women who reported use of SDNVP in a previous pregnancy had transmission rates that were similar to multiparous women who were drug naïve. Moreover, women who used SDNVP for two pregnancies during the study did not experience increased transmission rates in the second pregnancy, despite having demonstrable progression in their HIV disease as indicated by a decline in CD4 cell counts of nearly 100 cells/uL. In conjunction with the previous published studies from South Africa/Cote d'Ivoire [8] and Uganda [9], these data indicate that reuse of SDNVP may not be associated with a loss of efficacy.

Our observations suggest that persistent small pools of resistant mutants do not affect the efficacy of subsequent courses of SDNVP for later pregnancies. Resistant viral variants may be created and can remain detectable for as long as one year post-exposure [5-7]. But because they do not seem to interfere with the ability of nevirapine to prevent transmission in subsequent deliveries, we assume that they represent a small and insignificant fraction of maternal virus. Our findings are therefore concordant with a model in which SDNVP efficacy relies on the inhibition of the majority of circulating strains. However, such a mechanism is in contrast to treatment efficacy, which is compromised by resistance mutations if started within 6–18 months after SDNVP exposure [3,4,14].

The main limitation of this study is its small size. Power calculations indicate that we had sufficient power (> 80% with two-tailed alpha of 5%) to detect a difference in transmission rates of 14% for the comparison between women (Table 1). While these data therefore cannot be seen as definite evidence *per se*, the cumulative evidence suggests that SDNVP will remain efficacious independent of subtype and geographic region.

The mean time between deliveries in our study of 22 months is comparable to the difference observed in the two preceding studies [8,9]. Our ability to assess the efficacy of second dosing of SDNVP during a subsequent but more closely spaced pregnancy is limited. However, detection and fading of nevirapine resistance is similar regardless of whether nevirapine exposure is repeated or for the first time [15,16].

## Conclusion

SDNVP does not appear to lose it's efficacy for preventing mother-to-child transmission when repeat dosing occurs during subsequent pregnancies in HIV-infected women in Zambia. SDNVP is likely to remain an effective prevention method for Zambia and other resource-limited settings where more complex and efficacious regimens [17] have not yet become available.

## Abbreviations

AOR: adjusted odds ratio; ARV: antiretroviral treatment; BMI: body mass index; CI: confidence interval; IQR: inter quartile range; PMTCT: prevention of mother-to-child HIV transmission; SD: standard deviation; SDNVP: single dose nevirapine; ZEBS: Zambia Exclusive Breastfeeding Study.

## Competing interests

The authors declare that they have no competing interests.

## Authors' contributions

All authors participated in the collection of the data, their analysis and interpretation, as well as in revising the final manuscript. GMA was responsible for all the laboratory aspects of the study. LK and JW designed and conducted the analysis, JW drafted the manuscript.

## Pre-publication history

The pre-publication history for this paper can be accessed here:


